# A comparison of calcium sources for ion-trap loading via laser ablation

**DOI:** 10.1007/s00340-025-08521-z

**Published:** 2025-07-21

**Authors:** Daisy R. H. Smith, Silpa Muralidharan, Roland Hablützel, Georgina Croft, Klara Theophilo, Alexander Owens, Yashna N. D. Lekhai, Scott J. Thomas, Cameron Deans

**Affiliations:** 1 National Quantum Computing Centre, Oxfordshire, OX11 0QX UK; 2 Present Address: Nu Quantum Ltd., Cambridge, UK

## Abstract

Trapped-ion technology is a leading approach for scalable quantum computing. A key element of ion trapping is reliable loading of atomic sources into the trap. While thermal atomic ovens have traditionally been used for this purpose, laser ablation has emerged as a viable alternative in recent years, offering the advantages of faster and more localized loading with lower heat dissipation. Calcium is a well-established ion for qubit applications. Here we examine a range of calcium sources for ablation and provide a comprehensive analysis of each. We consider factors such as ease of use, temperature and yield of the ablation plume, and the lifetime of ablation spots. For each target, we estimate the number of trappable atoms per ablation pulse for a typical surface and 3D ion trap.

## Introduction

Quantum computing is an emerging technology with the potential to transform industries such as pharmaceuticals and manufacturing [1, 2]. Trapped-ion quantum computing is a promising approach, with state of the art single- and two-qubit fidelities, high above the error correction threshold [3–7]. Trapped ions offer advantages as qubits due to their atomic uniformity and all-to-all connectivity. These properties have led to numerous proposals for scalable architectures [7–11].

Several different ions can be used for quantum computing, including $^{40}\text {Ca}^{+}$ and $^{43}\text {Ca}^{+}$, which exhibit high fidelity in both single- and two-qubit gates across various schemes [12–16], as well as high state-preparation and measurement fidelity [17]. Mixed-species interactions and sympathetic cooling have been successfully demonstrated with $^{9}\text {Be}^{+}$ and $^{88}\text {Sr}^{+}$ [18, 19] and the required lasers for Doppler cooling are readily available solid-state lasers, not requiring frequency doubling.

Ion traps have traditionally been loaded using atomic ovens, which resistively heat sources to release atoms into the trapping zone [20, 21]. This approach has several drawbacks, including slow ion-loading, coating of the ion-trap electrodes, generation of magnetic fields, and high thermal load applied to the trap [22, 23]. Another approach is ablation, where a pulsed laser creates a small atom plume from a target. Ablation offers several advantages, including a more localized source, faster and more precise timing of ion loading, and reduced thermal load applied to the ion trap [22–27].

A further advantage of ablation loading is the ability to use compound targets, which are often easier and cheaper to procure than pure elemental sources. Targets vary in key characteristics such as surface reflectivity and density which can affect the performance of ablation [24]. Previously, a range of targets have been compared for $^{88}\text {Sr}^{+}$ [22], $^{138}\text {Ba}^{+}$ [28], and pure calcium has been compared with $\text {CaTiO}_{3}$ [27].

Here, we present a study of six potential calcium targets and compare them by ease of preparation and mounting in the vacuum chamber and analysis of the plume in terms of yield and temperature. The yield and temperature values are calculated via time-resolved spectroscopy experiments on the $\text {S}_{0}$ $\rightarrow$ $\text {P}_{1}$ first-stage photoionization transition, and used to estimate the number of trappable atoms in the plume for each target for a representative surface and 3D trap. We also present digital microscope images of the targets after ablation.

## Target selection and preparation

**Table 1:** Comparison of ablation targets and their preparation

Target	Structure	Adhesive	% Calcium (%)	Comments
Black calcite	Bulk crystal	Epoxy	33.6	Easy to mount
White calcite	Powder	Indium	39.6	Easy to mount
Pure calcium	Bulk metal	Indium	99.0	Oxidizes in ambient conditions
Calcium titanate	Powder	Indium	29.5	Difficult to mount
Calcium carbide	Bulk crystal	Epoxy	50.0	Disintegrates after thermal cycle
White calcite	Bulk crystal	N/A	13.7	Does not glue with epoxy and indium

Considerations when selecting the targets for comparison included the materials’ availability, robustness, stability under UHV and cryogenic conditions, as well as ease of mounting. Some materials are prone to fracture or ignition at low temperatures, so careful consideration was given to avoid potential disturbances to pressure or temperature, which could affect the trapping of ions or subsequent experiments. Both bulk crystal and powdered targets were compared in terms of mounting and ablation performance.

Selected targets were mounted to the system using one of two adhesives: a vacuum-compatible epoxy (EPO-TEK H20E) or pure indium foil (thickness 0.1 mm, $\ge$ 99.995% trace metals basis), which was melted and applied as glue. The targets are fabricated with approximate dimensions of 10 mm x 5 mm, with varying thicknesses for each individual target. Considering the beam size and required spacing, this allows for around 350 ablation spots per target.

Table 1 provides a comparison of the various targets, detailing their mounting methods and preparation specifics, used to assess their suitability.

**Black calcite** (Si:CaCO_3_): Silicon-doped calcite crystals were bonded to the ablation mount using epoxy. This target is easy to mount and stable at room temperature and under vacuum conditions.

**White calcite** (CaCO_3_): White calcite powder was bonded to the ablation mount using indium. A thin layer of the powder was formed on the mount. The material remains stable at room temperature and under vacuum conditions. Bulk white calcite failed to adhere during gluing due to the high temperatures involved in both epoxy and indium bonding, which caused the crystals to fracture.

**Pure calcium (Ca)**: Pure calcium metal was selected for its high potential yield but requires careful handling under vacuum as it oxidizes in ambient conditions. Such targets may therefore need to be replaced when breaking the vacuum [27]. This motivates the use of chemically inert salts as alternatives and is particularly relevant when using expensive isotopically enriched targets. The target was mounted using indium, and initial handling can be performed in a glove box to prevent oxidation. Any oxide layer was removed by sanding and polishing the target surface. The target was then stored under vacuum prior to use.

**Calcium titanate **(CaTiO_3_): Calcium titanate did not bond effectively with the ablation mount, although a thin layer of the material was successfully applied for testing. The material remains stable at room temperature and under vacuum conditions.

**Calcium carbide **(CaC_2_): Calcium carbide, a bulk crystal, was bonded using epoxy. It required handling in a glove box to prevent oxidation or ignition during preparation. While stable at both room and cryogenic temperatures, heating and cooling caused the target to shatter, rendering it unsuitable for long-term use.

## Experimental setup

Ablation experiments were conducted in a vacuum chamber equipped with cryogenic cooling. The chamber is initially evacuated to a pressure of around $10^{-6}$ mbar and cooled to 3.3 K over a 24-hour period. Cryo-pumping results in a pressure below $10^{-11}$ mbar at the experimental region.

**Fig. 1:**
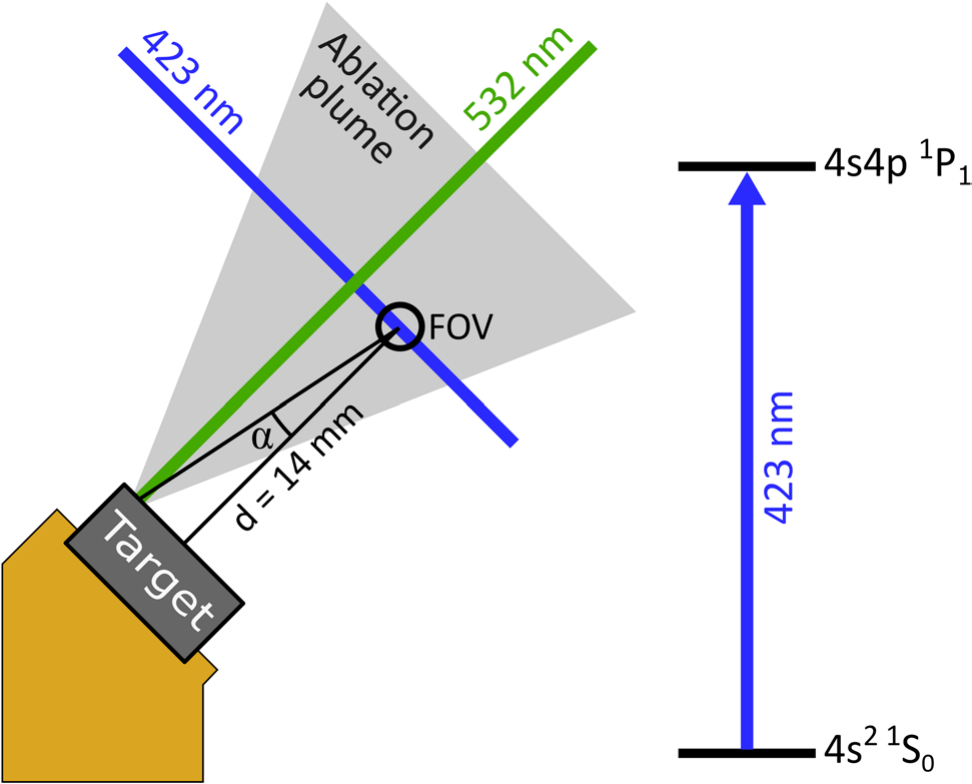
The experimental setup, showing the 532 nm (ablation) beam, the 423 nm (fluorescence) beam. FOV (field of view) refers to the area visible to the PMT. In reality this region is smaller than the diameter of the 423 nm beam. The angle $\alpha$ is the angle that an atom in the ablation plume makes with the normal to the fluorescence beam and *d* is the distance between the target surface and the FOV. The relevant calcium transition is shown on the right

The target is mounted on a gold-plated copper removable mount that is subsequently mounted inside the experimental region of the vacuum chamber. This allowed for fast changing of different test materials and easy replacement of targets. Ablation is performed using a 532 nm Nd:YAG pulsed laser (Bright Solutions WEDGE HB), and fluorescence detection is performed with a 423 nm CW laser (Toptica MDL Pro HP). The laser setup is illustrated in Fig. 1. Fluorescence from the ablation plume is detected by a photomultiplier tube (PMT).

The 532 nm laser has a pulse duration of 1.1 ns and a maximum pulse energy of 1 mJ. It is focused onto the target using an external lens mounted on an XYZ translation stage, allowing precise movement and control over the ablation spot. The spot is approximately 400 µm. Band pass filters at 423 nm are placed before the PMT to eliminate unwanted scattering, ensuring that only the fluorescence at 423 nm is detected. The 423 nm laser beam, with a diameter of 1.8 mm, is continuously applied. The laser wavelength is tuned to the resonant wavelength of the $4\text {s}^{2}$ $^{1}\text {S}_{0}$ $\rightarrow$ $4\text {s}4\text {p}^{1} \text {P}_{1}$ transition in ^40^Ca ($\lambda _\text {vac} = 422.7920$ nm) [29]. The power is chosen such that the intensity is significantly higher than 10 times the saturation intensity. The fluence of the 532 nm laser is gradually increased until the first ablation signal is detected. The fluence at which fluorescence is first detected is defined as the ablation threshold. Each target showed a different ablation threshold, as shown in Table 2. These differences are attributed to the different bond structures (ionic or metallic bonds) and the different material properties (powder or bulk crystal) of the targets.

## Data analysis

**Fig. 2:**
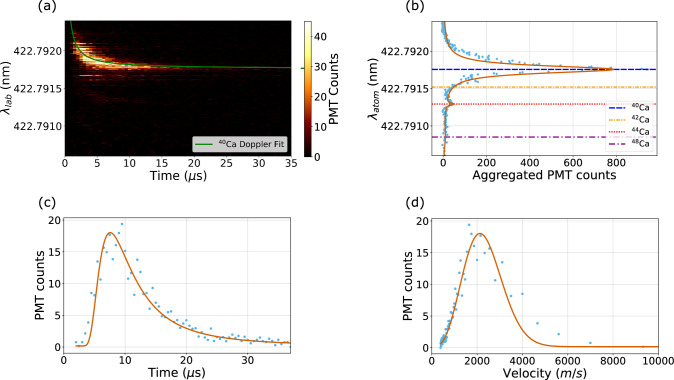
An example to show how the data for an ablation experiment was analyzed to obtain temperature and yield. This data was taken with the calcium carbide target. **a** Fluorescence counts scanned over time and wavelength ($\lambda _{lab}$). The data is fitted with Eq. 1 for angle ($\alpha$) and resonant wavelength ($\lambda _{atom}$) of the photoionization transition for ^40^Ca. **b** The sum of PMT counts along the hyperbola (Eq. 1) for a given wavelength, fitted with Eq. 2. **c** A time-of-flight distribution generated by the fluorescence along the hyperbola in **a**, fitted for temperature with a Maxwell–Boltzmann distribution curve (Eq. 4). **d** The atom velocity distribution, derived from **c**, included for illustrative purposes

Time-resolved spectroscopy was used to compare the temperature and yield of the ablation plume across the different targets. The experimental procedure involves setting a fixed wavelength of the 423 nm laser, triggering a pulse from the 532 nm laser, and measuring the fluorescence from the 423 nm photoionization transition over a 35 $\upmu$s interval using a PMT with a binning of 0.5 $\upmu$s. The process is repeated across a range of wavelengths, forming a plot such as that seen in Fig. 2a.

The data is used to calculate the angle that the ablation plume makes with the normal to the fluorescence beam, via fitting the equation

1 $$\begin{aligned} \lambda _{lab}(\lambda _{atom}, \alpha, t) = \lambda _{atom} \left( 1 - \frac{d \sin (\alpha )}{c \, t }\right), \end{aligned}$$

where $\lambda _{lab}$ is the wavelength of the incident 423 nm laser, $\lambda _{atom}$ is the wavelength of the ^40^Ca $\text {S}_{0}$ $\rightarrow$ $\text {P}_{1}$ transition in the atom frame, $\alpha$ is the angle between the atom’s trajectory and the normal to the fluorescence beam as seen in Fig. 1, *t* is the time since the ablation laser pulse, *d* is the distance from the center of the field of view and the surface of the ablation target (14 mm), and *c* is the speed of light in a vacuum. ^40^Ca is assumed to produce the highest fluorescence, as it has the highest natural abundance of 96.9% [21]. Fitting Eq. 1 to the brightest pixel per wavelength, determines $\alpha$ and $\lambda _{atom}$ for ^40^Ca, as seen in Fig. 2a.

Given $\alpha$ (which is fixed for all isotopes and a given spot), one can construct from Eq. 1, the hyperbola of data point which correspond to a given $\lambda _{atom}$. We sum along these hyperbola to produce Fig. 2b. This data is fitted with a sum of Lorentzians, one for each of the four most abundant calcium isotopes [21]

2 $$\begin{aligned} L_{\text {total}}(\lambda ) = \sum _{i=0}^{3} K_i \cdot \frac{\frac{s_i \cdot \gamma _i}{2}}{1 + s_i + \left( \frac{2\delta }{\gamma _i} \right) ^2} \,, \end{aligned}$$

where $\delta$ is the detuning from the resonant frequency of the $\text {S}_{0}$ $\rightarrow$ $\text {P}_{1}$ transition, $s_i$, $\gamma _i$, and $K_i$ are respectively the saturation parameter, linewidth, and scaling factor for isotopes ^40^Ca, ^42^Ca, ^44^Ca, and ^48^Ca. ^40^Ca consistently appears as the most abundant isotope, with ^44^Ca, which has the second-highest abundance (2.09%), typically observed as a minor peak. The other isotopes, which have abundances below 1% [21], are not clearly detected.

To determine the temperature of the ablation plume, we plot the time values and corresponding fluorescence along the ^40^Ca hyperbola (Eq. 1) from Fig. 2a, generating the time-of-flight data shown in Fig. 2c. This is then fitted with a Maxwell–Boltzmann distribution. As the processed data is generated by accounting for the Doppler shift, which only takes into account atom movement in one direction, a one-dimensional Maxwell–Boltzmann distribution,

3 $$\begin{aligned} \text {MB}(v) = A \exp \left( \frac{- m \, v^2}{2 \, k_B T }\right)\,, \end{aligned}$$

is used. The corresponding equation for time-of-flight is derived (see Appendix A) as

4 $$\begin{aligned} \text {MB}(t) = -A \frac{d}{t^3} \exp \left( \frac{- m \, d^2}{2 \, k_B t^2 T} \right), \end{aligned}$$

and is used to fit Fig. 2c to obtain a temperature value. The geometry of the system and dimensions of the targets are such that $\alpha$ is small ($\arccos {(\alpha )}\approx 1$). Therefore, we fix $d=$14 mm throughout to simplfy the analysis. The corresponding Maxwell–Boltzmann distribution over velocity is also shown for illustrative purposes in Fig. 2d.

## Results

The data analysis process as described in the previous section was conducted for all ablation spots on each target. Consistent with other literature [26], the number of successful spots (identified by the successful fitting of a clear fluorescence curve for ^40^Ca) varied across targets. The spots that produced clear fluorescence curves were collated to obtain the following yield and temperature results.

Yield and plume temperature are key metrics for comparing ablation targets. A high yield indicates a dense population of calcium atoms, increasing the likelihood of successful trapping. A low plume temperature corresponds to a higher population of slower atoms, which are easier to trap. This is particularly relevant in surface traps, which typically have a lower trap depth than 3D traps [30, 31].

Yield was determined by summing the total fluorescence and subtracting the background fluorescence in the time-resolved spectroscopy plots. The background fluorescence was determined by averaging the photon counts from the top-right quadrant of the time-resolved spectroscopy plot, an area that consistently showed no atomic signal.

**Fig. 3:**
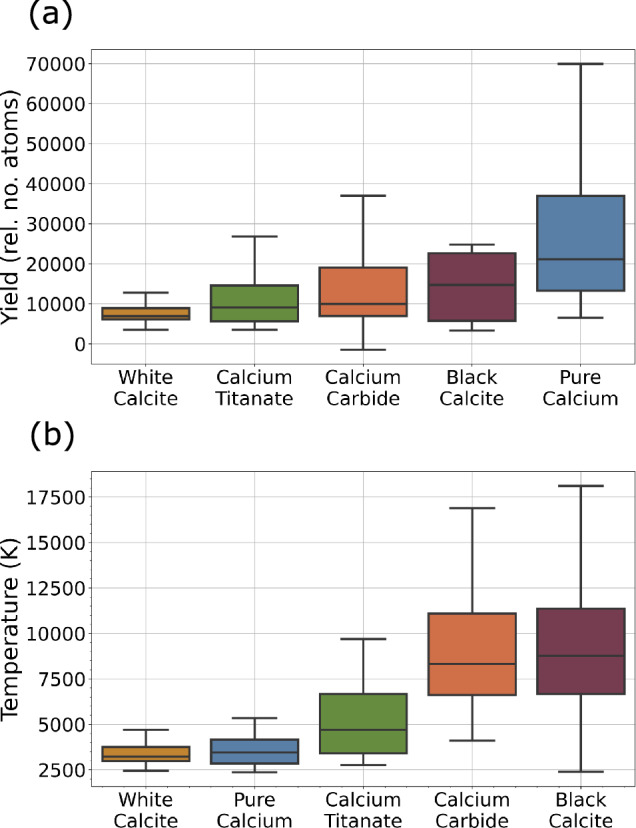
**a** Box-and-whisker plot presenting the spread of ablation plume yield for each target. **b** Plot presenting the spread of ablation plume temperature for each target

The results for yield are shown in Fig. 3a. The box-and-whisker plot shows that pure calcium exhibits the highest yield of 21000, while the two powders, white calcite and calcium titanate, show the lowest yields, with medians of 6900, and 9100 respectively. This is in-keeping with what we expect given metallic calcium is the most calcium-rich target and the bulk samples have lower surface roughness.

**Table 2:** Comparison of ablation targets and results

Target	Threshold (mJ/cm^2^)	Temperature (K)	Yield (rel. no. atoms)
White calcite	0.028	3200 [3200, 3400]	6900 [6700, 7800]
Pure calcium	0.013	3500 [2900, 3700]	21000 [14000, 31000]
Calcium titanate	0.028	4700 [4200, 5900]	9100 [7600, 10000]
Calcium carbide	0.059	8300 [7800, 8600]	10000 [9000, 13000]
Black calcite	0.108	8800 [7900, 9100]	15000 [6000, 22000]

Threshold refers to the fluence at which the ablation pulse first showed fluorescence. The temperatures and yield values are the median temperature and yield for each target as seen in Fig. 3a and b

The temperature of the ablation plume was calculated using a Maxwell–Boltzmann distribution fit, as discussed in Sect. 4. A box-and-whisker plot displays the results for each of the targets in Fig. 3b. It can be seen that white calcite and pure calcium produced the plumes with the lowest temperatures, with medians of 3200 K, and 3500 K. The temperatures for pure calcium and calcium titanate are similar to previously reported values (2696 K and 4211 K, respectively [27]).

**Fig. 4:**
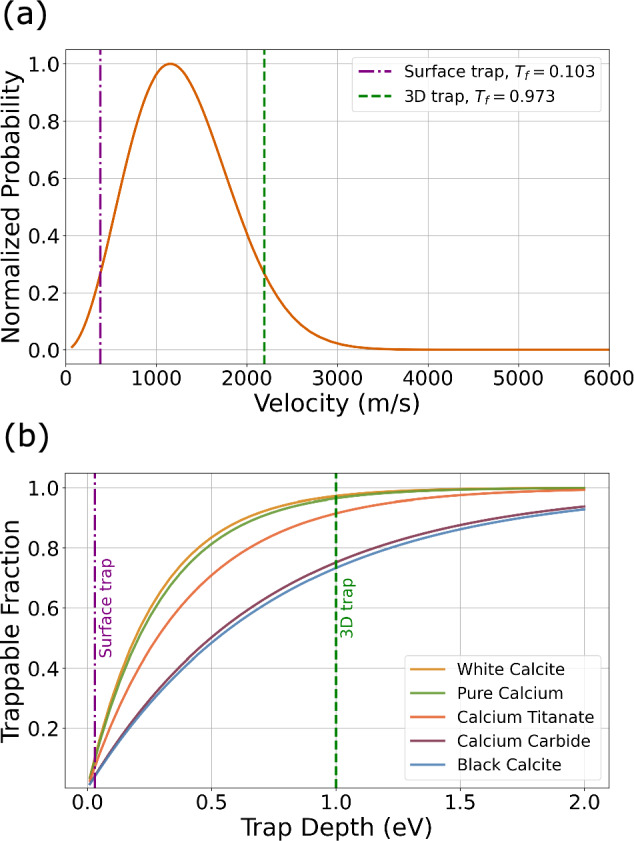
**a** Extrapolated Maxwell–Boltzmann distribution curve for white calcite, used to determine the fraction of trappable atoms in the ablation plume using the trap-depth for an example 3D trap and surface trap. $T_{f}$ is the trappable fraction of the atoms. **b** Trappable fraction of the atoms as a function of trap depth for all the targets. The vertical lines highlight the trap depths corresponding to our representative surface and 3D traps

Using the calculated median temperature for each target, we estimate the fraction of trappable atoms for a surface and 3D ion trap. Example trap-depths of 30 meV and 1 eV were chosen for a surface and 3D trap, respectively [30, 31]. The maximum velocity of a trappable atom was calculated from the trap depth, and the fraction of atoms below that velocity was established using the temperature (see Appendix C). The results are shown in Fig. 4a for white calcite, which had the lowest temperature and therefore the highest fraction of trappable atoms. The results for all targets are displayed in Table 3. To investigate the effect that variations in trap depths have on the trappable fraction we compute the trappable fraction as a function of trap depth. This was done for all the targets and is shown in Fig. 4b.

The fraction of trappable atoms was used to estimate a total number of trappable atoms for each target as described in Appendix C. The results are displayed in Table 3. For surface traps, pure calcium and white calcite had the highest estimated trappable atoms, while for 3D traps, the best targets were pure calcium and black calcite. This reflects the greater influence of temperature in surface traps, whereas in 3D traps, because the trap depth is higher, the yield of the target is of higher importance.

**Table 3:** Estimated fraction of trappable atoms in a typical ablation plume for a typical surface and 3D ion trap, determined using the median plume temperature for each target

Target	Fraction trappable		Estimated total trappable atoms	
	Surface trap	3D trap	Surface trap	3D trap
White calcite	0.103	0.97	42	400
Pure calcium	0.096	0.97	120	1200
Calcium titanate	0.072	0.92	38	490
Calcium carbide	0.041	0.75	24	440
Black calcite	0.039	0.73	34	650

The total number of trappable atoms is calculated from fluorescence-based estimates of the plume’s atomic population (see Appendix C)

**Fig. 5:**
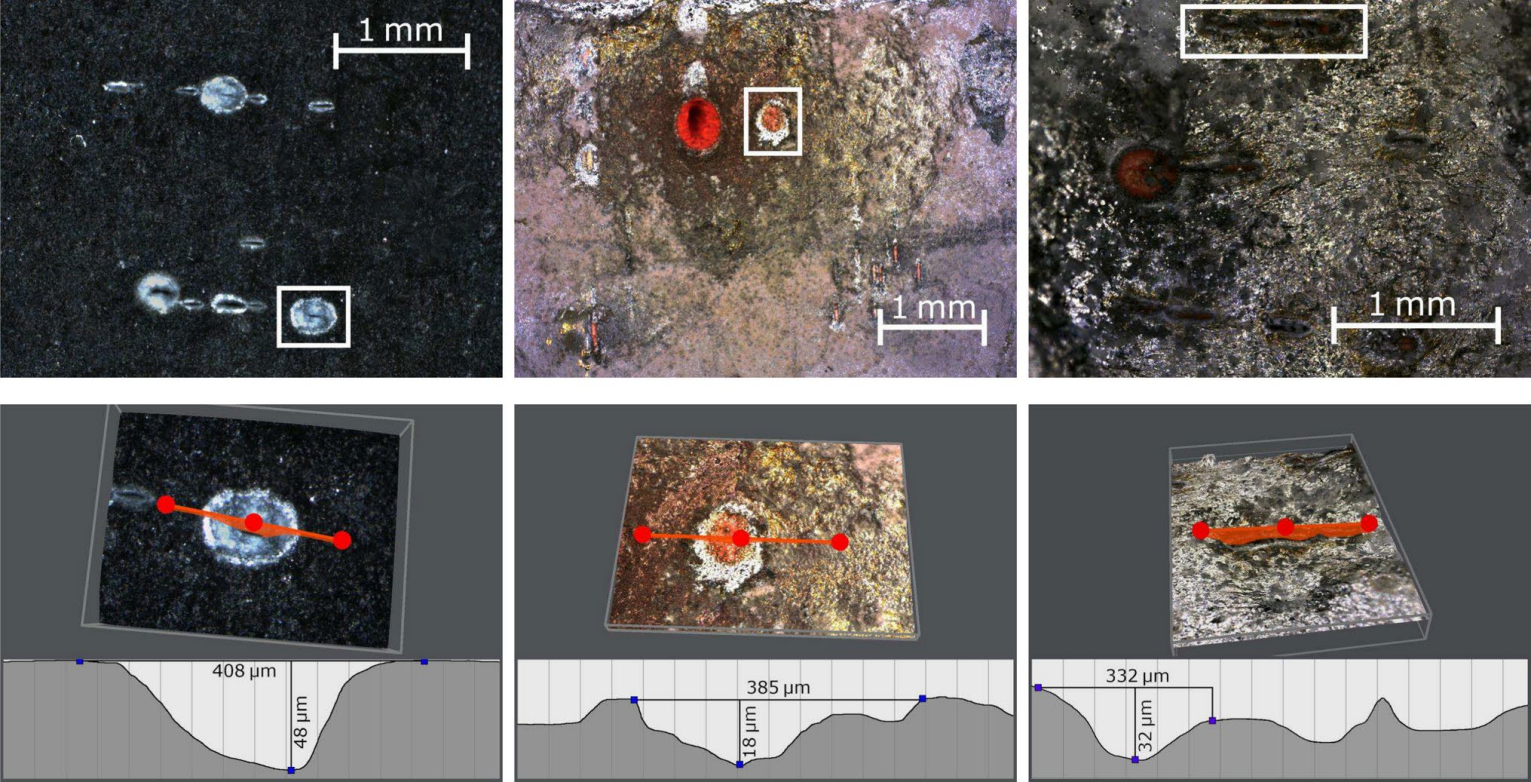
Images taken using a digital microscope to examine target-surfaces after ablation. Left panels: black calcite (bulk crystal). Middle panels: calcium titanate (powder). Right panels: white calcite (powder)

Another parameter of interest is spot lifetime, defined as the number of ablation pulses a target spot can endure before it becomes unusable, requiring realignment of the ablation beam to a new location. The primary factor influencing this parameter is whether the target is a powder or a bulk crystal. This likely reflects the preparation of the target and not its chemical properties. For the powders the lifetime was established by determining the point at which indium (the mount-target bonding agent) became visible. This was evident through an increase in the fluorescence across the entire time-resolved spectroscopy plot, which made the Doppler-shift hyperbola harder to detect (see Appendix B). This is attributed to strong resonance lines of singly-ionized indium at at 423 nm [32]. The lifetime of white calcite and calcium titanate were thus seen to be, on average, 1900 and 850 pulses respectively. This allows around 5 ×$10^5$ loading attempts before the sample is exhausted.

The lifetime of the bulk crystals were not directly observed. However, 4500, 6300, and 3000 pulses were applied for calcium carbide, black calcite, and pure calcium, respectively, without exhausting the ablation spot.

It should also be noted that the powder-spots were more inconsistent. For white calcite and calcium titanate there was a respective 31.8% and 30.4% probability of seeing no fluorescence at all from a spot, whereas for the bulk crystals 100% of the spots showed fluorescence. We attribute this to the thinner and more uneven surface of the powdered targets, as seen in Fig. 5, reducing the likelihood that a given spot consists of a sufficiently thick layer of the calcium target for reliable successful ablation.

**Fig. 6:**
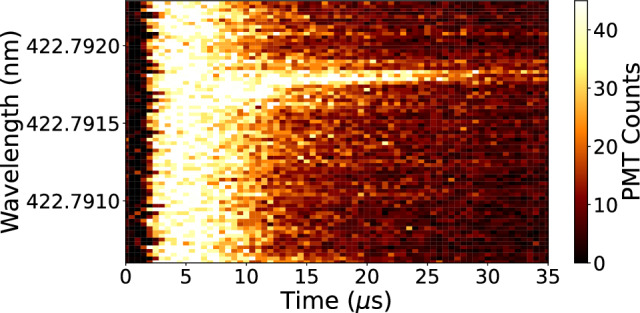
Example time-resolved spectroscopy plot where the ablation spot has been eroded down to the indium bonding material. The fluorescence counts are too high to distinguish the Doppler-shift hyperbola accurately. This is attributed to the presence of resonant lines of singly-ionized indium

A digital microscope (Evocam II) was used to examine the targets after ablation. The width and depth of the ablation spots were measured for black calcite, white calcite and calcium titanate. Post-ablation imaging of the calcium carbide and pure calcium targets was not possible, as the targets crumbled and oxidized, respectively, upon removal from the cryogenic vacuum system. Examples of the images obtained are shown in Fig. 5. The bulk crystal (black calcite) displays clear circular craters, whereas the powders show a more uneven surface around the ablation spots. This is attributed to the less rigid structure of the pasted powders as compared to the solid material. Figure 5 shows that the crater widths and depths are of the same order of magnitude across targets. The widths are attributed to the beam size, and therefore appear similar between targets. The red color of the ablation spots on the powder targets is due to the underlying copper oxide becoming exposed (Fig. 6).

## Conclusion

Ablation loading of ion traps has several advantages relevant to a range of quantum technologies applications. These include reduced thermal loads, localized atom sources, and fast precisely timed loading. We examined six potential ablation targets for calcium ion-trap loading. Black calcite and white calcite were found to be the most practical for UHV mounting. Ablation was performed with black calcite, white calcite powder, pure calcium, calcium titanate, and calcium carbide. Examination of the spot-lifetime showed that powders had shorter lifetimes than bulk crystals. Digital microscope images and analysis of bulk crystal and powder targets highlight the factors contributing to this discrepancy. Time-resolved spectroscopy was used to analyze the yield and temperature of the ablation plumes. Black calcite and pure calcium produced the highest yield, while white calcite and pure calcium had the lowest temperature. This was reflected in the estimation of the number of trappable atoms per ablation plume, where pure calcium and white calcite were preferable for surface traps, while pure calcium and black calcite were preferable for 3D traps. However, the preparation environment and exposure duration of a particular experiment may eliminate pure calcium due to oxidation. The target may have to be cleaned with a series of high-power pulses to remove the oxide layer following each system modification [28] or replaced entirely [27]. In this case, we conclude that white calcite powder would be the most suitable target material for surface traps and black calcite would be the most suitable for 3D traps. This extends to other species of fast-oxidizing metals, such as strontium and barium, and is of particular importance when considering expensive isotopically enriched targets.

## Data Availability

No datasets were generated or analysed during the current study.

## Appendix A: Derivation of the Maxwell–Boltzmann time-of-flight fitting equation

We consider only one dimension because the processed data accounts for the Doppler shift which only applies to the direction of the laser propagation. The standard Maxwell–Boltzmann distribution equation for velocity in one dimension is

A1 $$\begin{aligned} \text {MB}(v) = A \exp \left( \frac{- m \, v^2}{2 \, k_B T }\right)\,, \end{aligned}$$

were *m* is the mass of the atom, *T* is the temperature of the ablation plume, $k_B$ is the Boltzmann constant, and *A* is a scaling factor. We use the chain rule to rearrange the equation in terms of time-of-flight

A2 $$\begin{aligned} {\text {MB}(t) = \text {MB}(v) \times \frac{dv}{dt} \,,} \end{aligned}$$

A3 $$\begin{aligned} \frac{dv}{dt} = \frac{d}{dt} \left[ \frac{d}{t}\right] = -\frac{d}{t^2} \,, \end{aligned}$$

where *d* represents the distance between the target and the PMT’s field of view, and *t* denotes the time-of-flight, the duration taken by the atoms to travel from the target to the field of view.

Slower atoms contribute more to the fluorescence signal as they spend more time transiting through the imaging system’s field of view (FOV). To account for this contribution, we correct with a factor $\frac{1}{t}$ (proportional to the atom’s velocity) and get

A4 $$\begin{aligned} \text {MB}(t) = -A \frac{d}{t^3} \exp \left( \frac{- m \, d^2}{2 \, k_B t^2 T} \right), \end{aligned}$$

which is used to fit the time-of-flight data for a temperature value in Sect. 4.

## Appendix B: Example of a time-resolved spectroscopy plot with indium fluorescence

When a powder spot was depleted, fluorescence from indium dominated the signal. This is attributed to strong resonant lines of In II at 423 nm [32]. Upon removing the targets from the vacuum for inspection, the ablation spot was found to have eroded down to the indium layer, but not the copper beneath, confirming indium as the fluorescence source.

## Appendix C: Trappable atoms for all targets

The maximum velocity of a trappable atom is calculated using equation

C1 $$\begin{aligned} v_{\text {max}} = \sqrt{\frac{2E_{\text {trap}}}{m}} \,, \end{aligned}$$

where $E_{\text {trap}}$ is the trap depth and *m* is the mass of a calcium atom. From $v_{\text {max}}$ the minimum time-of-flight for trappable atoms can be calculated, and thus the fraction of trappable atoms is determined via

C2 $$\begin{aligned} T_{f} = \frac{\int _{\text {TOF}_{\min }}^{\infty } \frac{d}{t^3} \exp \left( -\frac{m d^2}{2 t^2 k_B T}\right) dt}{\int _{0}^{\infty } \frac{d}{t^3} \exp \left( -\frac{m d^2}{2 t^2 k_B T}\right) dt} \,. \end{aligned}$$

The maximum velocity of a trappable atom is shown on a Maxwell–Boltzmann distribution for each target, generated using the median temperature calculated for each target.

Given the estimated collection efficiency of the imaging set-up ($5\%$, based on the solid-angle calculation and estimation of the beam path efficiency to the PMT) and the PMT’s quantum efficiency (31%), we estimate the overall photon-collection efficiency as $\frac{P_{PMT}}{P_A} = 0.02$, where $P_{PMT}$ is the number of photons collected by the PMT and $P_A$ is the total number of photons produced by the atom cloud within the FOV.

Using a representative atom velocity of 2000 ms^−1^, the scattering rate of the 423 nm transition is approximately 220 MHz [29]. With an estimated excited-state population of 40%, each atom scatters photons at a rate of $s_{r} = 0.4 \times {220\,\mathrm{\text {M}\text {Hz}}} = {88\,\mathrm{\text {M}\text {Hz}}}$. Over the fluorescence duration, the total number of scattered photons is given by $t \times s_{r} = {0.9\,\mathrm{\upmu \text {s}}} \times {88\,\mathrm{\text {M}\text {Hz}}} = 79$ photons per atom. This results in about 0.01 atoms per photon at the atom cloud. We calculate the number of atoms per photon seen at the PMT to be

C3 $$\begin{aligned} \frac{A}{P_{PMT}} = \frac{A}{P_{A}} \times \frac{P_{A}}{P_{PMT}} = 0.01 \times \frac{1}{0.02} = 0.5 \,, \end{aligned}$$

where *A* is the number of atoms in the atom cloud, $P_{PMT}$ is the total number of photons collected by the PMT, $P_A$ is the total number of photons produced by the atom cloud.

To estimate the total number of trappable atoms, the median yield for each target was used to scale a typical time-resolved spectroscopy plot so that its total fluorescence matched the median yield. The brightest row in the plot was then assumed to represent the total fluorescence from the ablation plume when illuminated near resonance, for a single ablation pulse. This was multiplied by $\frac{A}{P_{PMT}}$ to estimate the number of atoms in the ablation plume, and then by the trappable fraction ($T_{f}$) to estimate the total number of trappable atoms. Error ranges are calculated using the quartiles of the corresponding yield and temperature data. The results are seen in Table 4.

**Table 4:** Number of trappable atoms from the ablation plume, for a single laser pulse, for each target type

Target	Fraction trappable	Number of atoms	Total trappable
*Surface ion trap*			
White calcite	0.103	406	42 [36, 45]
Pure calcium	0.096	1234	118 [99, 143]
Calcium titanate	0.072	535	38 [27, 52]
Calcium carbide	0.041	587	24 [18, 30]
Black calcite	0.039	882	34 [27, 45]
*3D ion trap*			
White calcite	0.973	406	395 [387, 397]
Pure calcium	0.965	1234	1191 [1159, 1214]
Calcium titanate	0.915	535	489 [441, 517]
Calcium carbide	0.751	587	442 [381, 486]
Black calcite	0.733	882	646 [564, 727]

Calculated using the trap depth of a typical surface ion trap and a typical 3D ion trap. Ranges calculated using the quartiles of the corresponding yield and temperature data
